# The Efficacy and Safety of Seladelpar for Primary Biliary Cholangitis: A Systematic Review and Meta‐Analysis

**DOI:** 10.1002/jgh3.70265

**Published:** 2025-08-28

**Authors:** Mohamed Abuelazm, Saqr Alsakarneh, Mohammad Tanashat, AlMothana Manasrah, Ahmed A. Ibrahim, Sandesh Parajuli, Hatem Eltaly, Ahmed Mazen Amin

**Affiliations:** ^1^ Faculty of Medicine Tanta University Tanta Egypt; ^2^ Gastroenterology and Hepatology Division Mayo Clinic Rochester Minnesota USA; ^3^ Internal Medicine Department United Health Services Wilson Medical Center Johnson City NY USA; ^4^ Faculty of Medicine Menoufia University Menoufia Egypt; ^5^ Department of Medicine Reading Hospital Reading Pennsylvania USA; ^6^ Cleveland Clinic Main Campus Hospital Cleveland Ohio USA; ^7^ Faculty of Medicine Mansoura University Mansoura Egypt

**Keywords:** biliary, drug, gallbladder, hepatology, liver, seladelpar

## Abstract

**Background and Objective:**

Seladelpar is an oral, once‐daily medication that improves cholestasis through its selective peroxisome proliferator‐activated receptor (PPAR‐δ) agonism. It shows promising efficacy in treating primary biliary cholangitis (PBC) patients.

**Methods:**

A systematic review and meta‐analysis synthesizing evidence from randomized controlled trials (RCTs) obtained from PubMed, Cochrane, Scopus, and WOS until July 19th, 2025. Dichotomous outcomes were reported using risk ratio (RR) and continuous outcomes using mean difference (MD), with a 95% confidence interval (CI).

**Results:**

Three RCTs with 499 patients were included. Seladelpar was significantly associated with an increased ALP normalization (RR: 21.12 with 95% CI [4.14, 107.58], *p* < 0.01), biochemical response (RR: 3.06 with 95% CI [2.00, 4.70], *p* < 0.01), and decreased pruritus NRS score change (MD: −1.47 with 95% CI [−2.73, −0.21], *p* = 0.02). Seladelpar was also significantly associated with a decreased incidence of pruritus (RR: 0.54 with 95% CI [0.31, 0.94], *p* = 0.03) but with an increased incidence of headache (RR: 3.37 with 95% CI [1.11, 10.23], *p* = 0.03). However, there was no significant difference between seladelpar and placebo regarding the incidence of any adverse events (RR: 0.96 with 95% CI [0.87, 1.06], *p* = 0.43).

**Conclusion:**

Seladelpar improved liver biomarkers of cholestasis and reduced pruritus in patients with PBC without significantly increasing the adverse effects. This makes seladelpar a promising addition to the treatments available for PBC.

**Trial Registration:** PROSPERO: CRD42024521208

## 1 Introduction

Primary biliary cholangitis (PBC) is an autoimmune disease characterized by chronic granulomatous inflammation of small intrahepatic ducts and the accumulation of toxic bile acids that may ultimately result in cirrhosis and liver failure [1]. PBC predominantly affects middle‐aged Caucasian females, but about one in every 10 PBC patients is male, and one in every 10 is non‐Caucasian [2, 3, 4]. It has a progressive clinical course, and approximately 20% of these patients will remain symptom‐free after 10 years of diagnosis—the majority of the patients without treatment progress to liver failure, transplantation, and death [5]. Ursodeoxycholic acid (UDCA) is the first line of treatment and is the only agent currently approved by the Food and Drug Administration (FDA). It works mainly by reducing ductal inflammation, improving liver enzyme levels, and decreasing the risk of liver transplantation or death [6]. However, about 40% of patients either do not tolerate or do not respond to UDCA. For patients with persistently elevated ALP, unchanged bilirubin levels, or signs of disease progression, obeticholic acid (OCA) is a second FDA‐approved option [7].

The limitation, however, is that pruritus has been present in patients treated with OCA, as seen in more than 65% of the patients treated with it [8]. Pruritus is not just common in PBC but also severely impacts patients' quality of life to the extent that it sometimes necessitates liver transplantation [9]. In some cases, fibrates like fenofibrate and bezafibrate are also used off‐label for their cholestatic property. Additionally, cholestyramine, sertraline, and antihistamines have been frequently used recently to mitigate pruritus [7]. Therefore, further FDA‐approved drugs remain warranted for PBC.

Seladelpar is an oral, once‐daily medication that improves cholestasis through its selective peroxisome proliferator‐activated receptor (PPAR‐δ) agonism [10]. It decreases bile acid transport in the cholangiocytes and downregulates cholesterol synthesis, a substrate for bile acid [11]. Seladelpar also has anti‐fibrotic and anti‐inflammatory action through Kupffer's and stellate cells [12]. This widespread effect of seladelpar in bile acid homeostasis has made it a potent option, and it has been studied in different clinical trials to treat PBC [13, 14]. Recently, the FDA issued an accelerated approval of seladelpar for PBC management after promising results from the RESPONSE trial [13, 15]. Therefore, we conducted a systematic review and meta‐analysis to comprehensively assess the current evidence on the efficacy and safety of seladelpar in patients with PBC.

## 2 Methodology

### 2.1 Protocol Registration

We followed PRISMA statement guidelines when reporting this systematic review and meta‐analysis [16]. Also, all steps were done per the Cochrane Handbook of Systematic Reviews and Meta‐analysis of Interventions (version 5.1.0) [17]. Under the following ID: CRD42024521208, this review has been registered and published in PROSPERO.

### 2.2 Data Sources and Search Strategy

We searched the following electronic medical databases: PubMed, SCOPUS, Web of Science, and CENTRAL from inception till July 19th, 2025, and we used the following research query: (seladelpar OR “PPAR‐delta agonist” OR “PPAR‐δ agonist”) AND (“primary biliary cholangitis” OR “primary biliary cirrhosis” OR PBC). The detailed search process of each database is presented in Table S1. No language restrictions were applied during the search or study selection to ensure comprehensiveness.

### 2.3 Eligibility Criteria

We included randomized controlled trials with the following PICO criteria: population (P), patients with PBC; intervention (I), seladelpar irrespective of the dosage; control (C), placebo; and outcomes (O), our primary outcomes: ALP normalization, biochemical response (defined as an ALP level less than 1.67 times the upper limit of the normal range (ULN), with a decrease of 15% or more from baseline, and a total bilirubin level up to 1.0 times the ULN), and the change in pruritus NRS score in patients with a baseline score > four. Our secondary outcomes included liver enzyme levels change, bilirubin change, pruritus scale change, quality of life scale changes, and adverse events.

### 2.4 Study Selection

Records were imported to Covidence for duplicate detection and exclusion. Three authors (A.M., M.T., and M.A.) blindly screened the articles based on title and abstract. Then, full‐text screening was conducted, followed by the extraction phase of the articles that met the eligibility criteria. In the case of a conflict, the third author (M.A.) resolved it.

### 2.5 Data Extraction

Three reviewers (A.M., M.T., and A.A.I.) employed a pilot‐tested extraction sheet to extract the following data from the included RCTs: summary of included studies (name of the first author, publication year, study design, blinding, country of the study, total number of participants, seladelpar dose, frequency of administration, treatment duration, main inclusion criteria, primary outcome, and follow up duration); baseline information (number of patients in each group, age, male, basal metabolic index (BMI), disease duration, pruritus history, AMA positive, cirrhosis, pruritus NRS score, and baseline liver enzymes); and study outcomes as previously described. Conflicts were resolved by discussion among reviewers to attain consensus.

### 2.6 Risk of Bias and Certainty of Evidence

Three reviewers (A.M., M.T., and A.A.I.) independently performed the quality assessment of the included studies using the Risk of Bias 2 (RoB2) tool [18], and M.A. resolved discrepancies. The following domains were evaluated individually: randomization process, deviations from intended intervention, missing outcome data, measuring the outcomes, and selecting reported results. Any discrepancies were settled through discussion between the reviewers.

To appraise the quality of evidence, two reviewers (M.A.) utilized the Grading of Recommendations Assessment, Development, and Evaluation (GRADE) guidelines [19, 20]. We considered inconsistency, imprecision, indirectness, publication bias, and risk of bias. The evaluation was carried out for each outcome, and the decisions were justified and documented.

### 2.7 Statistical Analysis

Statistical analyses were conducted using Review Manager v.5.4 [21]. We estimated the mean difference (MD) for continuous outcomes and risk ratios (RR) along with the 95% confidence interval (CI). We used the random‐effects model if heterogeneity was found, as it accommodates a more significant standard error in the pooled estimate, making it suitable for inconsistent or controversial estimates. A *p* value ≤ 0.05 is considered statistically significant. If continuous variables were expressed as a median and interquartile range, the mean and standard deviation were computed based on the median, interquartile range, and sample size, as described by Hozo et al. [22]. Web Plot Digitizer was used to help reverse engineer numerical data from images of plots [23].

Regarding the heterogeneity, the chi‐square test evaluated statistical heterogeneity among studies. Then, the chi‐square statistic was used to calculate *I*‐squared. A chi‐square with a *p* value less than 0.1 was considered significant heterogeneity. Also, the *I*‐squared value of more than or equal to 50% indicated high heterogeneity. Additionally, we performed sensitivity analyses to determine the robustness of the effect size by removing one study at a time to check the strength of the evidence and ensure the overall results were not altered.

## 3 Results

### 3.1 Search Results and Study Selection

**FIGURE 1:**
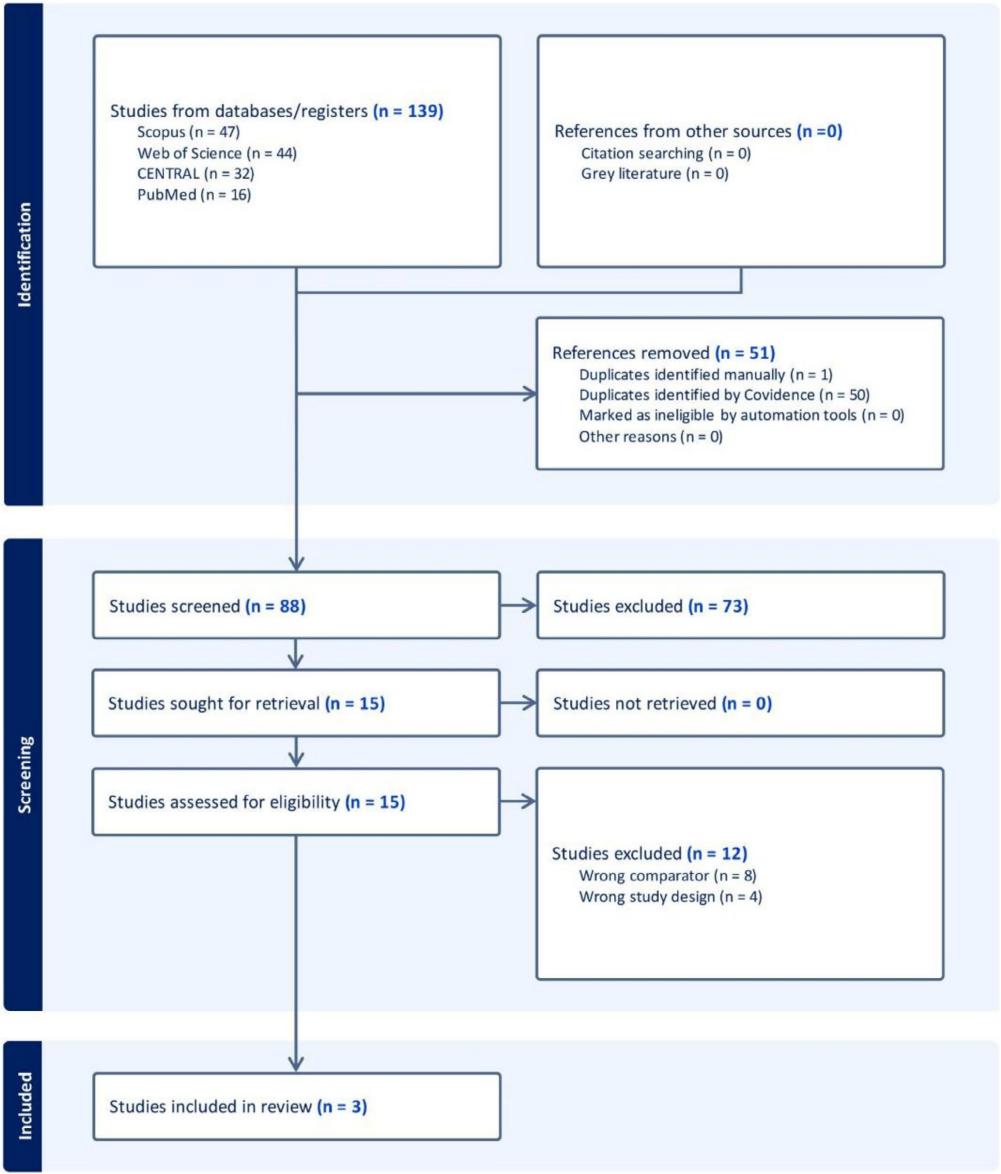
PRISMA flow chart of the screening process.

Our literature search retrieved 139 unique records. Fifty‐one records were removed as duplicates by Covidence. After title and abstract screening, 15 studies were eligible for full‐text screening. Finally, three studies were included in this systematic review and meta‐analysis. The PRISMA flowchart for study selection is shown in Figure 1.

### 3.2 Characteristics of Included Studies

Three RCTs were included in the meta‐analysis with 499 patients [10, 13, 14]. All the included studies assessed our primary outcome. The follow‐up duration in those studies ranged from 3 to 12 months. These studies were conducted in more than 24 countries. The summary and baseline characteristics of the included studies are shown in Tables 1 and 2, respectively.

**TABLE 1:** Summary characteristics of the included RCTs.

Study ID	Study design	Total participants	Seladelpar			Primary outcome	Follow‐up duration
			Dose	Frequency of administration	TTT duration		
Hirschfield et al. 2023 (ENHANCE)	Phase 3, double‐blinded, multicenter RCT	265	5 or 10 mg	Once daily	6 months	Composite biochemical response defined as ALP < 1.67 × ULN, ≥ 15% ALP decrease from baseline, and total bilirubin ≤ ULN at month 3.	6 months + follow‐up visit 4 weeks after the end of treatment
Hirschfield et al. 2024 (RESPONSE)	Phase 3, double‐blinded, multicenter RCT	193	10 mg	Once daily	12 months	Biochemical response: an alkaline phosphatase level less than 1.67 times the ULN, with a decrease of 15% or more from baseline, and a total bilirubin level up to 1.0 times the ULN.	12 months
Jones et al. 2017	Phase 2, double‐blinded, multicenter RCT	41	50 mg, or 200 mg	Once daily	3 months	Change from baseline in alkaline phosphatase concentrations over 12 weeks.	12 weeks

Abbreviations: ALP: alkaline phosphatase, RCT: randomized controlled trial, ULN: upper limit of the normal range.

**TABLE 2:** Baseline characteristics of the participants.

Study ID	Dose	Number of patients in each group		Age (years), mean (SD)		Male, *N* (%)		BMI, mean (SD)		Disease duration, mean (SD)		Pruritis history, *N* (%)		Antimitochondrial antibodies positive, *N* (%)	
		Seladelpar	Placebo	Seladelpar	Placebo	Seladelpar	Placebo	Seladelpar	Placebo	Seladelpar	Placebo	Seladelpar	Placebo	Seladelpar	Placebo
Hirschfield et al. 2023 (ENHANCE)	5 mg	89	87	54.7 (9.7)	55.9 (8.2)	7 (8)	2 (2)	27.7 (6.1)	28.2 (5.5)	8.3 (6.4)	8.4 (6.2)	66 (74)	57 (66)	79 (89)	75 (86)
	10 mg	89		55.6 (9.1)		6 (7)		27.6 (5.9)		8.4 (6.4)		65 (73)		81 (91)	
Hirschfield et al. 2024 (RESPONSE)	10 mg	128	65	56.6 ± 10.0	57.0 ± 9.2	5 (3.9)	5 (7.7)	NA	NA	8.2 ± 6.7	8.6 ± 6.5	91 (71.1)	48 (73.8)	NA	NA
Jones et al. 2017	50 mg	13	13	55 (10)	55 (10.8)	1 (8)	1 (8)	24 (5)	28 (6)	7.7 (8.3)	6 (6.6)	4 (31)	4 (33)	NA	NA
	200 mg	12	13	58.7 (9.2)	55 (10.8)	0	1 (8)	27 (4)	28 (6)	10.7 (17.6)	6 (6.6)	5 (50)	4 (33)	NA	NA

Study ID	Cirrhosis, *N* (%)		Pruritus NRS score				Baseline liver chemistries, mean (SD)							
	Seladelpar	Placebo	≥ 4—*N* (%)		≥ 4, mean (SD)		Alkaline phosphatase, IU/L		Aspartate aminotransferase, IU/L		Alanine aminotransferase, IU/L		Gamma‐glutamyl transpeptidase, IU/L	
			Seladelpar	Placebo	Seladelpar	Placebo	Seladelpar	Placebo	Seladelpar	Placebo	Seladelpar	Placebo	Seladelpar	Placebo
Hirschfield et al. 2023 (ENHANCE)	9 (10)	7 (8)	27 (30)	27 (31)	6.1 (1.4)	6.1 (1.2)	290.5 (104.2)	293.4 (106.2)	40.1 (14.5)	37.5 (16.8)	47.7 (21.0)	44.4 (20.7)	231.3 (212.0)	228.9 (193.0)
	13 (15)		27 (30)		6.2 (1.4)		290.8 (109.1)		40.3 (14.9)		46.9 (20.8)		243.1 (227.7)	
Hirschfield et al. 2024 (RESPONSE)	18 (14.1)	9 (13.8)	49 (38.3)	23 (35.4)	6.1 ± 1.4	6.6 ± 1.4	314.6 ± 123.0	313.8 ± 117.7	39.6 ± 16.1	41.7 ± 16.0	47.4 ± 23.5	48.2 ± 22.8	269.0 ± 240.0	287.5 ± 249.6
Jones et al. 2017	NA	NA	NA	NA	NA	NA	312 (95)	233 (73)	37 (18)	36 (12)	47 (31)	40 (24)	220 (152)	183 (123)
	NA	NA	NA	NA	NA	NA	248 (89)	233 (73)	32 (11)	36 (12)	32 (15)	40 (24)	104 (41)	183 (123)

Abbreviations: BMI, basal metabolic index; *N*, number; NA, not available; SD, standard deviation.

### 3.3 Risk of Bias and Certainty of Evidence

**FIGURE 2:**
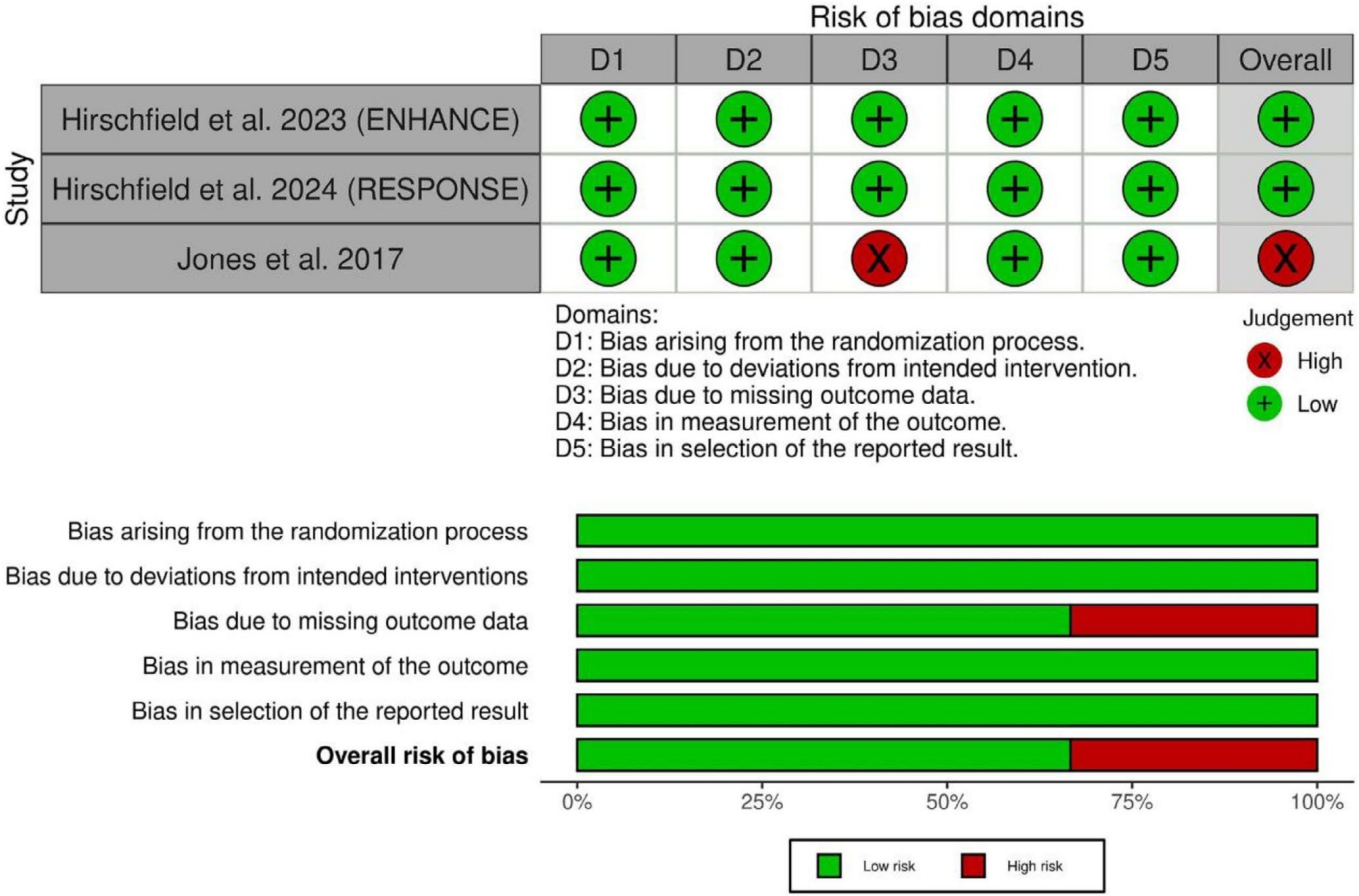
Quality assessment of risk of bias in the included trials. The upper panel presents a schematic representation of risks (low = green, unclear = yellow, and high = red) for specific types of biases of each study in the review. The lower panel presents risks (low = green, unclear = yellow, and high = red) for the subtypes of biases of the combination of studies included in this review.

The risk of bias assessment for each outcome is presented in Figure 2. Two included studies demonstrated a low risk of bias in all domains. However, Jones et al. had an overall high risk of bias, primarily from issues related to the missing outcome data due to a significant loss of follow‐up. The certainty of evidence is outlined in Table 3.

**TABLE 3:** GRADE evidence profile.

Certainty assessment						
Participants (studies) follow‐up	Risk of bias	Inconsistency	Indirectness	Imprecision	Publication bias	Overall certainty of evidence
ALP Normalization						
369 (3 RCTs)	Serious^a^	Not serious	Not serious	Serious^b^	Very strong association	⨁⨁⨁⨁ High
Biochemical Response						
262 (2 RCTs)	Not serious	Not serious	Not serious	Serious^b^	None	⨁⨁⨁◯ Moderate
Pruritus NRS Score Change						
77 (2 RCTs)	Not serious	Not serious	Not serious	Very serious^c^	None	⨁⨁◯◯ Low
ALP Change						
271 (3 RCTs)	Not serious	Not serious	Not serious	Serious^d^	Very strong association	⨁⨁⨁⨁ High
GGT Change						
236 (2 RCTs)	Not serious	Not serious	Not serious	Very serious^c^	Strong association	⨁⨁⨁◯ Moderate
ALT Change						
234 (2 RCTs)	Not serious	Not serious	Not serious	Very serious^c^	None	⨁⨁◯◯ Low
Pruritis						
496 (3 RCTs)	Not serious	Not serious	Not serious	Serious^e^	None	⨁⨁⨁◯ Moderate
Any Adverse Event						
496 (3 RCTs)	Not serious	Not serious	Not serious	Very serious^e^	None	⨁⨁◯◯ Low
Any Serious Adverse Events						
495 (3 RCTs)	Not serious	Not serious	Not serious	Very serious^e^	None	⨁⨁◯◯ Low

Abbreviation: CI: confidence interval.

^a^ Jones et al. showed a high risk of attrition bias.

^b^ Low number of events (< 300 events).

^c^ A wide confidence interval that does not exclude the risk of appreciable benefit/harm, with a low number of participants (< 400).

^d^ Low number of participants (< 400 participants).

^e^ A wide confidence interval that does not exclude the risk of appreciable benefit/harm, with a low number of events (< 300 events).

### 3.4 Primary Outcomes

**FIGURE 3:**
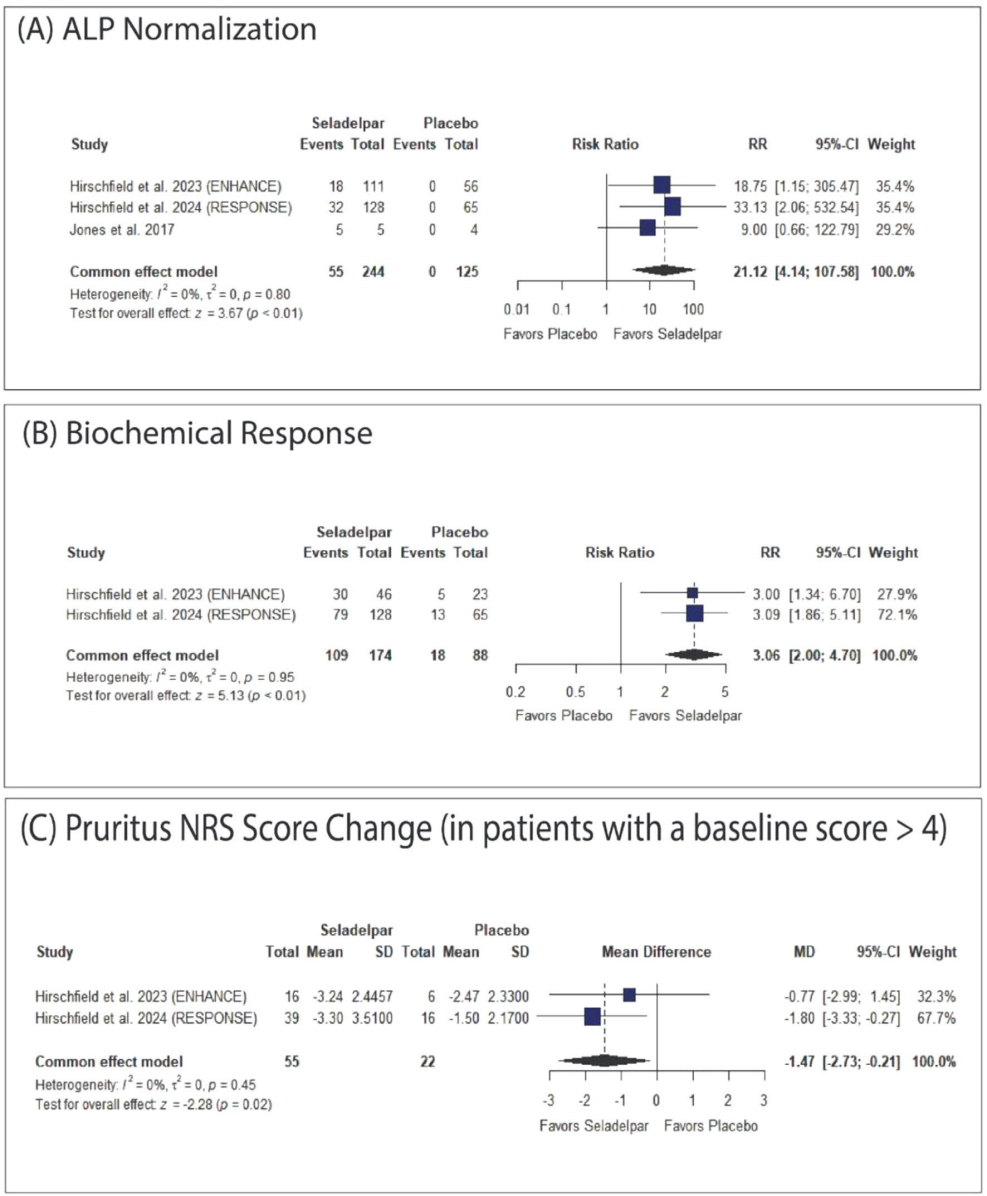
Forest plots of the primary outcomes. CI: confidence interval, MD: mean difference, RR: risk ratio.

Seladelpar was significantly associated with an increased ALP normalization (RR: 21.12 with 95% CI [4.14, 107.58], *p* < 0.01), biochemical response (RR: 3.06 with 95% CI [2.00, 4.70], *p* < 0.01), and decreased pruritus NRS score change (MD: −1.47 with 95% CI [−2.73, −0.21], *p* = 0.02) Figure 3.

The pooled studies were homogenous in ALP normalization (*I* ^2^ = 0%, *p* = 0.80), biochemical response (*I* ^2^ = 0%, *p* = 0.95), and pruritus NRS score change (*I* ^2^ = 0%, *p* = 0.45).

### 3.5 Secondary Outcomes

#### 3.5.1 Liver Enzymes

**FIGURE 4:**
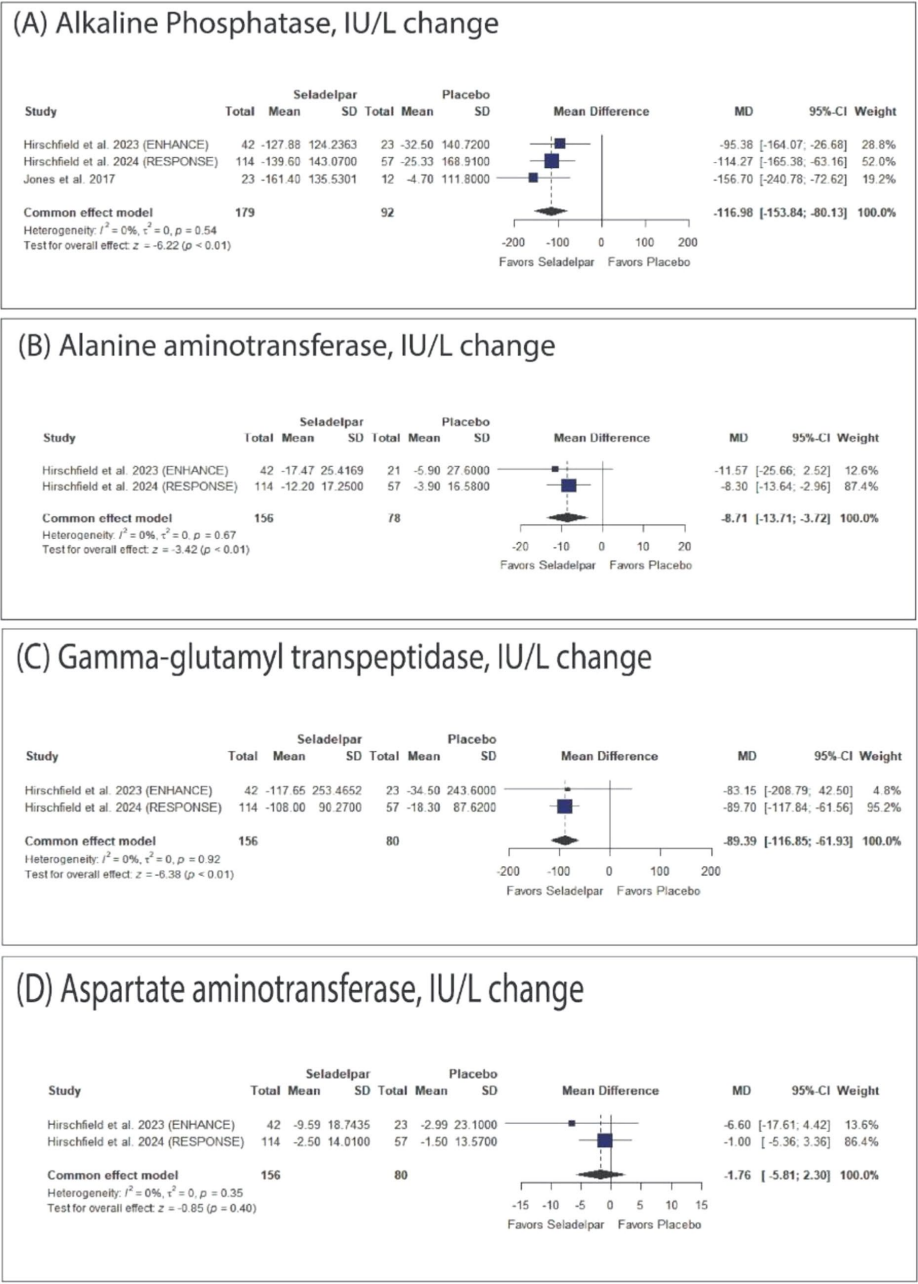
Forest plots of change in the liver enzymes. CI: confidence interval, MD: mean difference.

Seladelpar was significantly associated with decreased ALP (MD: −116.98 with 95% CI [−153.84, −80.13], *p* < 0.01), ALT (MD: −8.71 with 95% CI [−13.71, −3.72], *p* < 0.01), and GGT (MD: −89.39 with 95% CI [−116.85, −61.93], *p* < 0.01). However, there was no significant difference between seladelpar and placebo in AST (MD: −1.76 with 95% CI [−5.81, 2.30], *p* = 0.40) (Figure 4).

The pooled studies were homogenous in ALP (*I* ^2^ = 0%, *p* = 0.54), ALT (*I* ^2^ = 0%, *p* = 0.67), GGT (*I* ^2^ = 0%, *p* = 0.92), and AST (*I* ^2^ = 0%, *p* = 0.35).

#### 3.5.2 Safety Outcomes

**FIGURE 5:**
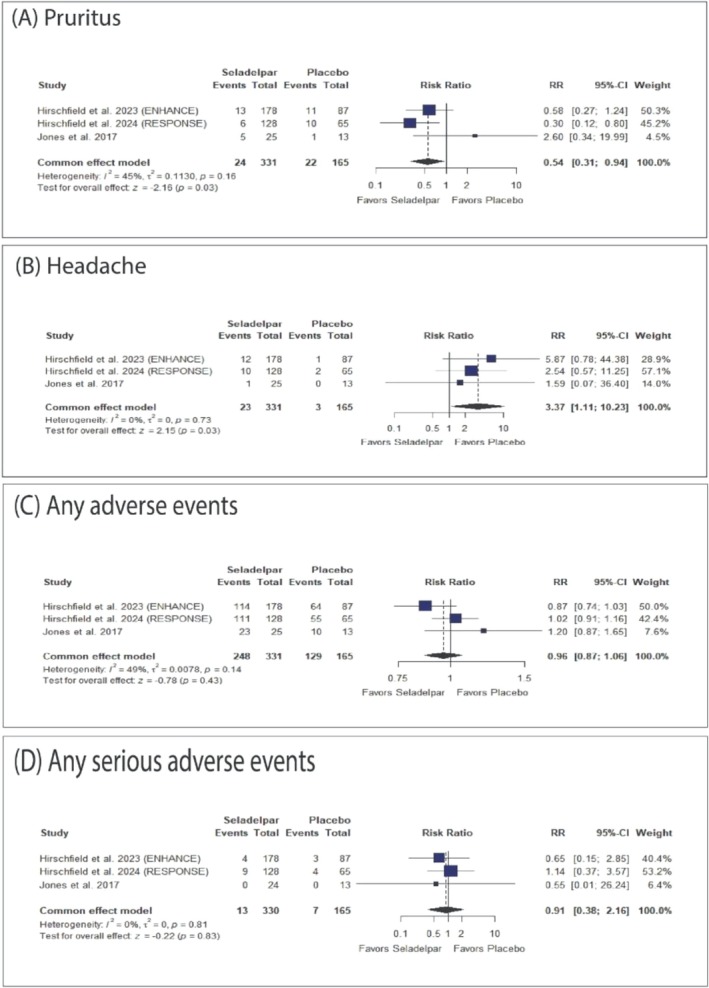
Forest plot of the safety outcomes. CI: confidence interval, RR: risk ratio.

Seladelpar was significantly associated with a decreased incidence of pruritus (RR: 0.54, 95% CI [0.31, 0.94], *p* = 0.03) (Figure 5A) and an increased incidence of headache (RR: 3.37, 95% CI [1.11, 10.23], *p* = 0.03) (Figure 5B). However, there were no significant differences between seladelpar and placebo in the incidence of any AEs (Figure 5C), any SAEs (Figure 5D), or other specific adverse events (Figures S1–S7).

The pooled studies were generally homogeneous across most outcomes, except for heterogeneity observed for URTs, for which sensitivity analyses were not applicable.

#### 3.5.3 Lipid Profile

Seladelpar was significantly associated with decreased LDL cholesterol (MD: −8.39 with 95% CI [−16.67, −0.11], *p* = 0.05) and TGs (MD: −18.11 with 95% CI [−30.05, −6.16], *p* < 0.01). However, there was no significant difference between seladelpar and placebo in HDL cholesterol (MD: 3.10 with 95% CI [−0.98, 7.18], *p* = 0.14) and total cholesterol (MD: −8.31 with 95% CI [−18.12, 1.50], *p* = 0.10) (Figure S8).

The pooled studies were homogenous in LDL cholesterol (*I* ^2^ = 0%, *p* = 0.88), TGs (*I* ^2^ = 0%, *p* = 1.00), HDL cholesterol (*I* ^2^ = 0%, *p* = 0.38), and total cholesterol (*I* ^2^ = 0%, *p* = 0.84).

#### 3.5.4 Other Parameters

Seladelpar was significantly associated with decreased C4 Change (MD: −6.26 with 95% CI [−11.68, −0.84], *p* = 0.02). However, there was no significant difference between seladelpar and placebo in total bilirubin, 5‐domains itch scale change, PBC‐40 quality‐of‐life questionnaire change, and IgM serum level change (Figures S9–S13). The pooled studies were homogenous except in the 5‐domains itch scale (*I* ^2^ = 72%, *p* = 0.06), but sensitivity analysis was not applicable.

## 4 Discussion

Our meta‐analysis showed that seladelpar significantly reduced alkaline phosphatase levels, increased biochemical parameters, and ameliorated pruritis without significantly increasing the risk of adverse effects. Wetten et al., in their review based on preliminary data, labeled seladelpar as an attractive second‐line agent for treating PBC [24]. Our study presents the most recent data on seladelpar treating PBC.

Bile acid homeostasis is a function of hepatocytes, cholangiocytes, and intestinal cells. Different PPAR receptors are present in these cells, but PPAR‐δ has the advantage of being expressed in all these cells and is an effective target for therapy [25]. Lin et al. studied four types of PPAR agonists and highlighted the significant effect of reducing ALP levels [26]. Similarly, in a different study, Elafibranor, a mixed PPAR‐α and δ agonist, was found to have a significant anti‐inflammatory effect and decreased ALP level [27]. Our study is consistent with the findings in these studies on different PPAR agonists and provides evidence regarding seladelpar, a PPAR‐δ agonist.

PPAR‐δ, through its expression in the Kupffer and stellate cells, downregulates the hepatic inflammation cascade. Our study shows a significant decrease in the C4 complement level. As proposed in the study by Honda et al., inhibition of tumor necrosis factor‐alpha and interleukin‐1 alpha is associated with immunomodulation affecting C4 complement levels [28]. However, this improvement in the inflammatory marker did not statistically improve fatigue in our analysis.

Moreover, bile acid is a pruritogen and is an essential cause of pruritus, the most debilitating symptom of PBC [29]. It is understood that around 65% of PBC patients have pruritus at some point in their course, and approximately a third of them have persistent itchiness [30]. All studies included in our analysis reported improvement in pruritus, and our study showed a significant MD of −1.47 in the pruritus score. Improvement in pruritus with seladelpar in PBC was reported recently and attributed to decreased bile acids and interleukin‐31 [31]. Therefore, it may be speculated that seladelpar may be used for pruritic amelioration, regardless of ALP normalization, warranting further study.

Also, patients with PBC often have co‐existing metabolic diseases, including hypercholesterolemia [32]. Our study showed the beneficial effect of seladelpar on lipid profile by decreasing LDL cholesterol and triglyceride levels. A similar finding was observed when seladelpar was used alone or in combination with other agents like liraglutide when studied in non‐alcoholic steatohepatitis, where a decrease in triglyceride and total cholesterol was seen in an animal model [33]. This finding is promising in decreasing the atherogenic effects associated with PBC, which may increase the cardiovascular risk in patients with metabolic risk factors [34].

Trials involving seladelpar were stopped due to safety concerns before. This has been addressed with a change in the dosage, and after independent pathologists reviewed liver histology, trials were resumed [24]. A phase III double‐blind 12‐month trial on seladelpar at a reduced dose of 10 mg daily concluded without reporting such issues, highlighting the safety of this medication [13]. However, an increased incidence of headaches was noticed in our study. Burra et al. discussed alteration in cerebral blood flow in patients with liver disease as a possible etiology of this side effect [35]. Furthermore, upper respiratory tract infection (URTI) was seen in a few patients, but significant heterogeneity was observed in the URTI report. Interestingly, headache and nasopharyngitis were also seen in 19% and 28% of the patients receiving OBA for PBC [36]. Overall, our study's safety profile of seladelpar appeared favorable, with no significant increase in adverse events or serious adverse effects. As most trial participants received background UDCA, our findings primarily reflect seladelpar's additive effects. Its efficacy as monotherapy in UDCA‐intolerant/non‐responders remains to be established, highlighting a critical area for future research.

After proving its efficacy and safety, further research to put seladelpar in PBC management guidelines remains warranted. A recent network meta‐analysis investigating pharmacological interventions for UDCA‐resistant PBC management concluded that among second‐line therapies, PPAR agonists demonstrated superior efficacy in improving ALP biochemical levels compared to drugs with alternative mechanisms, with elafibranor exhibiting the highest efficacy, followed by saroglitazar [26]. However, they included only the ENHANCE trial of seladelpar [14]. Thus, their findings remain preliminary and warrant further confirmation. Head‐to‐head RCTs are warranted to investigate the most effective PPAR agonist as a second line for PBC management.

## 5 Strengths and Limitations

This is the first systematic review and meta‐analysis investigating the safety and efficacy of seladelpar in patients with PBC, constituting the gold‐standard evidence. However, our study has a few limitations. The sample size of the included trials was small, and due to a lack of sufficient phase III studies, a phase II study was also included in our study. Similarly, the funnel plot was also not constructed to assess publication bias. Also, most patients in all three trials were simultaneously receiving UDCA; thus, the benefit attributed to seladelpar should be understood more as an add‐on therapy than a monotherapy. Additionally, while our focus on RCTs strengthens internal validity, the exclusion of observational studies may limit generalizability to patients with advanced disease or comorbidities, who are often underrepresented in trials. Future reviews could consider including high‐quality observational data to address this gap. The GRADE assessment indicated high certainty for ALP normalization and moderate‐to‐low certainty for secondary outcomes (e.g., pruritus improvement). These ratings underscore the need for larger, long‐term trials to confirm findings, particularly for safety endpoints where evidence remains less robust (Table 3). Finally, the long‐term safety of seladelpar could not be addressed because of the lack of long‐term data beyond 12 months.

## 6 Implications for Future Research

Seladelpar has shown anti‐inflammatory action through the Kupffer cells and the stellate cells. How much of this can translate into delaying fibrosis and progression to cirrhosis is yet to be known. More extensive clinical trials with extended follow‐up periods will help understand the long‐term safety and efficacy of seladelpar. Also, testing the safety of seladelpar in patients with compensated cirrhosis and hepatic impairment is another step to be addressed in other RCTs. Finally, further studies are warranted to investigate the prospect of seladelpar as a monotherapy in patients who fail to respond or are intolerant of UDCA, besides head‐to‐head comparisons with other approved second‐line drugs.

## 7 Conclusion

Seladelpar improved liver biomarkers of cholestasis and reduced pruritus in patients with PBC without significantly increasing the adverse effects. Seladelpar demonstrates promise as an add‐on therapy for PBC, with significant improvements in cholestasis and pruritus. However, the small number of trials and participants warrants cautious interpretation until further large‐scale studies confirm these results. It should be noted clearly that the observed effects of seladelpar are indicative of its use as an adjuvant treatment rather than as a stand‐alone intervention, as the majority of patients included in the trials were receiving background UDCA therapy.

## Ethics Statement

The authors have nothing to report.

## Consent

The authors have nothing to report.

## Conflicts of Interest

The authors declare no conflicts of interest.

## Supporting information


**Table S1:** Search strategy.
**Figures S1–S7:** Forest plots of adverse events.
**Figure S8:** Forest plots of lipid profile.
**Figure S9:** Forest plot of C4 change.
**Figure S10:** Forest plot of total bilirubin change.
**Figure S11:** Forest plot of 5‐domains itch scale change.
**Figure S12:** Forest plot of PBC‐40 quality‐of‐life questionnaire change.
**Figure S13:** Forest plot of IgM serum level change.

## Data Availability

The data that support the findings of this study are available from the corresponding author upon reasonable request.
