# Supplementary material for: The Efficacy and Safety of Seladelpar for Primary Biliary Cholangitis: A Systematic Review and Meta‐Analysis
Source: JGH Open. 2025 Aug 28;9(9):e70265. doi: 10.1002/jgh3.70265 (PMC12394184; doi:10.1002/jgh3.70265)
Supplement: Supplementary file 1 — Table S1: Search strategy. Figures S1–S7: Forest plots of adverse events. Figure S8: Forest plots of lipid profile. Figure S9: Forest plot of C4 change. Figure S10: Forest plot of total bilirubin change. Figure S11: Forest plot of 5‐domains itch scale change. Figure S12: Forest plot of PBC‐40 quality‐of‐life questionnaire change. Figure S13: Forest plot of IgM serum level change. [file JGH3-9-e70265-s001.docx]

**Supplementary Materials:**

**Title.**

**The Efficacy and Safety of Seladelpar for Primary Biliary Cholangitis: A Systematic Review and Meta-Analysis.**

**Running Title.**

Seladelpar for Primary Biliary Cholangitis.

**Authors.**

Mohamed Abuelazm-MBBCh^1,*^, Saqr Alsakarneh-MD^2,*^, Mohammad Tanashat-MBBCh^3^, AlMothana Manasrah-MBBCh^3^, Ahmed A. Ibrahim-MBBCh^4^, Sandesh Parajuli-MD^5^, Hatem Eltaly-MD^6^, Ahmed Mazen Amin-MBBCh^7^

**Affiliations.**

1. Faculty of Medicine, Tanta University, Tanta, Egypt.
2. Department of Internal Medicine, University of Missouri-Kansas City, Kansas, USA.
3. Faculty of Medicine, Yarmouk University, Irbid, Jordan.
4. Faculty of Medicine, Menoufia University, Menoufia, Egypt.
5. Department of Medicine, Reading Hospital, Reading, PA, USA.
6. Cleveland Clinic Main Campus Hospital, Cleveland, Ohio, USA.
7. Faculty of Medicine, Mansoura University, Mansoura, Egypt.

*, Both authors have equal contributions and are co-first authors.

**Keywords.**

Seladelpar; cirrhosis; cholangitis; drug; review; analysis.

**Contents:**

**Tables.**Table S1: Search strategy.

**Figures**.

Figures S1-7: Forest plots of adverse events.

Figure S8: Forest plots of lipid profile.

Figure S9: Forest plot of C4 change.

Figure S10: Forest plot of total bilirubin change.

Figure S11: Forest plot of 5-domains itch scale change.

Figure S12: Forest plot of PBC-40 quality-of-life questionnaire change.

Figure S13: Forest plot of IgM serum level change.

| Database | Search Terms | Search Field | Search Results |
| --- | --- | --- | --- |
| Pubmed | (seladelpar OR “PPAR-delta agonist” OR “PPAR-δ agonist”) AND (“primary biliary cholangitis” OR “primary biliary cirrhosis” OR PBC) | All Fields | 16 |
| Cochrane | (seladelpar OR “PPAR-delta agonist” OR “PPAR-δ agonist”) AND (“primary biliary cholangitis” OR “primary biliary cirrhosis” OR PBC) | All Text | 32 |
| WOS | (seladelpar OR “PPAR-delta agonist” OR “PPAR-δ agonist”) AND (“primary biliary cholangitis” OR “primary biliary cirrhosis” OR PBC) | All Fields | 44 |
| Scopus | (seladelpar OR “PPAR-delta agonist” OR “PPAR-δ agonist”) AND (“primary biliary cholangitis” OR “primary biliary cirrhosis” OR PBC) | Title, Abstract, Keywords | 47 |

Table S1: Search Strategy.


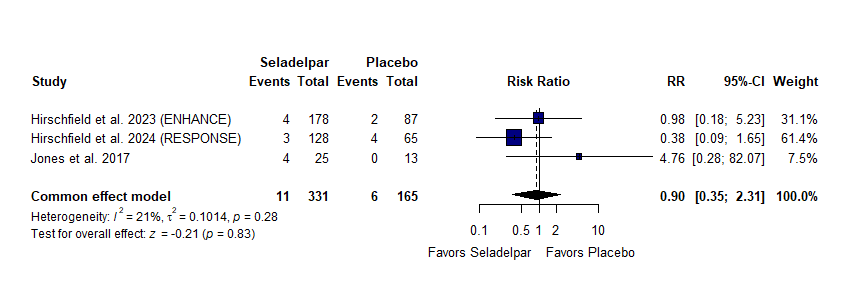


Figure S1: Any adverse event leading to drug withdrawal.


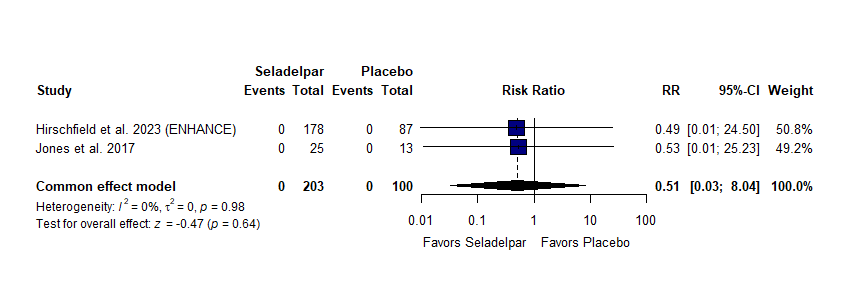


Figure S2: All-cause mortality.


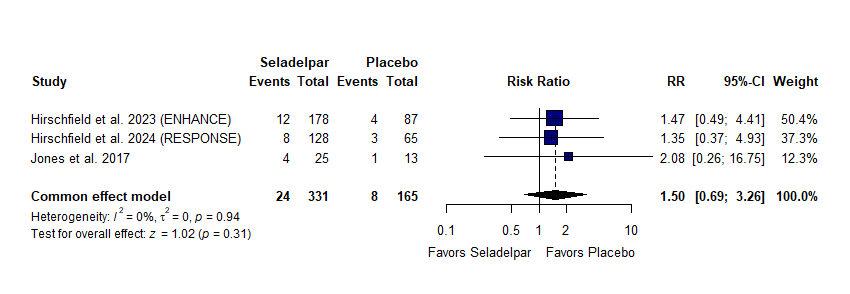


Figure S3: nausea.


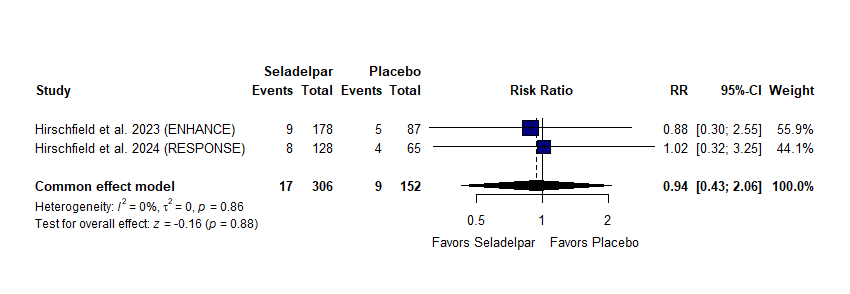


Figure S4: arthralgia.


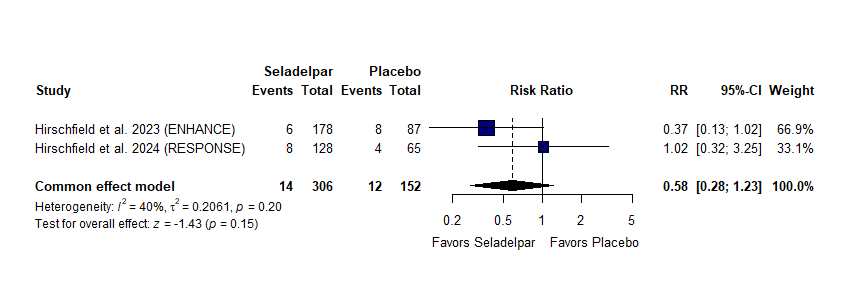


Figure S5: fatigue.


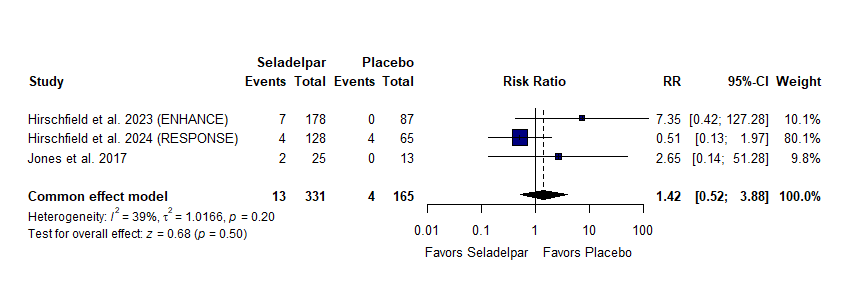


Figure S6: UTIs.


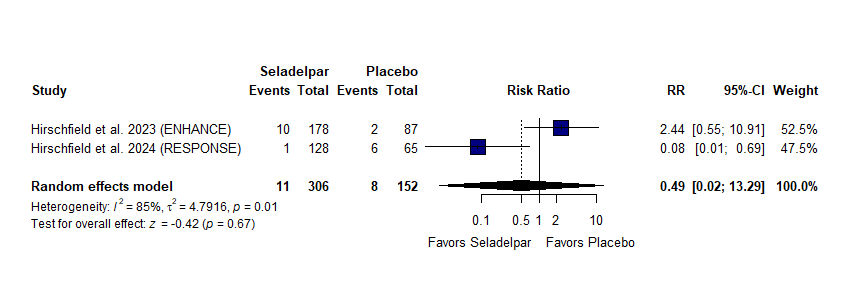


Figure S7: URTIs.


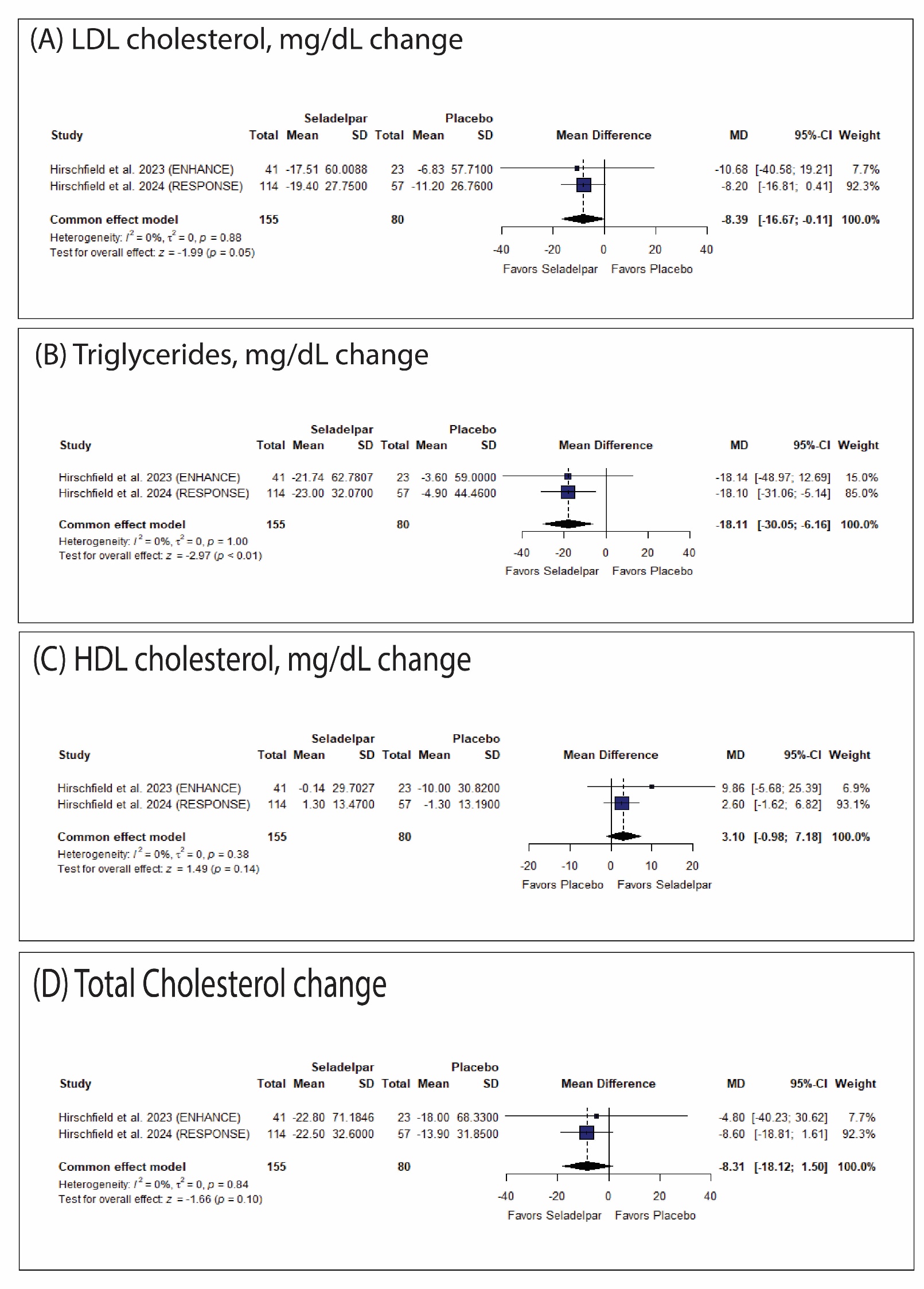


Figure S8: Lipid profile.


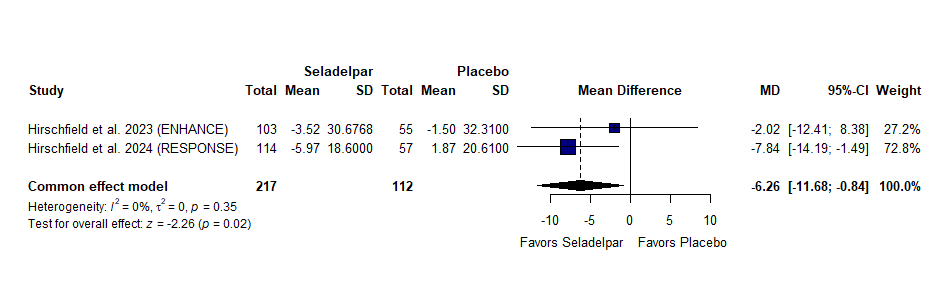


Figure S9: C4 change.


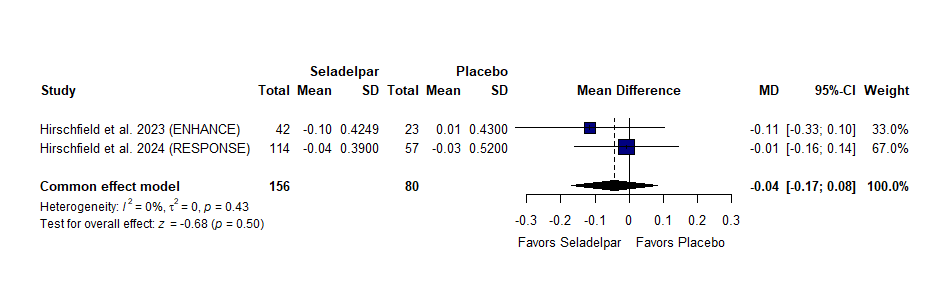


Figure S10: Forest plot of total bilirubin change.


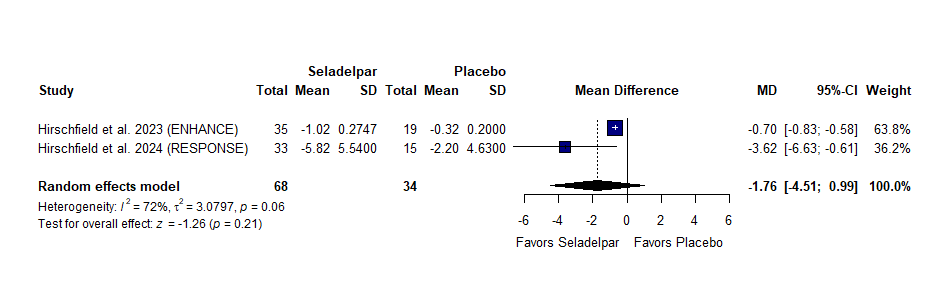


Figure S11: Forest plot of 5-domains itch scale change.


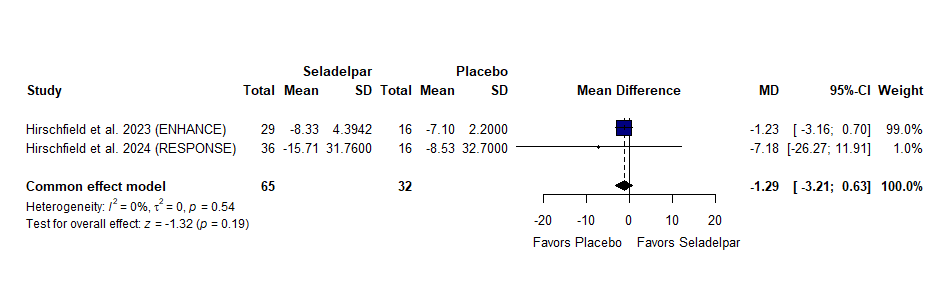


Figure S12: Forest plot of PBC-40 quality-of-life questionnaire change.


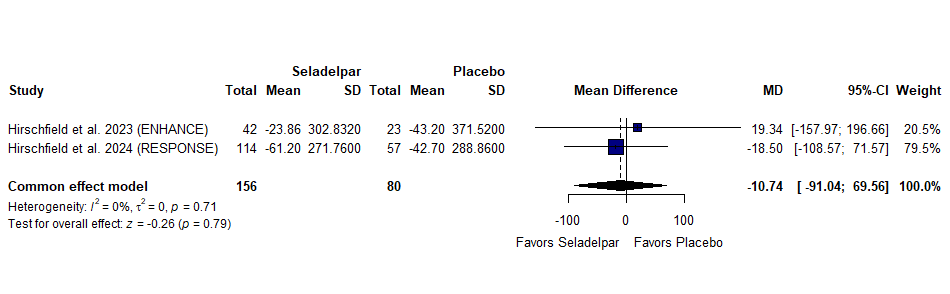


Figure S13: Forest plot of IgM serum level change.
